# Peripheral Endocannabinoid Responses to Hedonic Eating in Binge-Eating Disorder

**DOI:** 10.3390/nu9121377

**Published:** 2017-12-20

**Authors:** Alessio Maria Monteleone, Fabiana Piscitelli, Riccardo Dalle Grave, Marwan El Ghoch, Vincenzo Di Marzo, Mario Maj, Palmiero Monteleone

**Affiliations:** 1 Department of Psychiatry, University of Campania Luigi Vanvitelli, 80138 Naples, Italy; alessio.monteleone@fastwebnet.it (A.M.M.); majmario@tim.it (M.M.); 2 Endocannabinoid Research Group, Institute of Biomolecular Chemistry, Consiglio Nazionale delle Ricerche, 80078 Pozzuoli, Naples, Italy; fpiscitelli@icb.cnr.it; 3 Department of Eating and Weight Disorders, Villa Garda Hospital, 37016 Garda, Verona, Italy; rdalleg@gmail.com (R.D.G.); marwan1979@hotmail.com (M.E.G.); 4 Neuroscience Section, Department of Medicine, Surgery and Dentistry “Scuola Medica Salernitana”, University of Salerno, 84081 Salerno, Italy

**Keywords:** anandamide, binge-eating disorder, 2-arachidonoylglycerol, endocannabinoids, hedonic eating

## Abstract

Reward mechanisms are likely implicated in the pathophysiology of binge-eating behaviour, which is a key symptom of binge-eating disorder (BED). Since endocannabinoids modulate food-related reward, we aimed to investigate the responses of anandamide (AEA) and 2-arachidonoylglycerol (2-AG) to hedonic eating in patients with BED. Peripheral levels of AEA and 2-AG were measured in 7 obese BED patients before and after eating favorite (hedonic eating) and non-favorite (non-hedonic eating) foods. We found that plasma levels of AEA progressively decreased after eating the non-favorite food and significantly increased after eating the favorite food, whereas plasma levels of 2-AG did not differ significantly between the two test conditions, although they showed a trend toward significantly different time patterns. The changes in peripheral AEA levels were positively correlated to the subjects’ sensations of the urge to eat and the pleasantness while eating the presented food, while changes in peripheral 2-AG levels were positively correlated to the subjects’ sensation of the pleasantness while eating the presented food and to the amount of food they would eat. These results suggest the occurrence of distinctive responses of endocannabinoids to food-related reward in BED. The relevance of such findings to the pathophysiology of BED remains to be elucidated.

## 1. Introduction

In the last edition of the Diagnostic and Statistical Manual of Mental Disorders (DSM-5) [1] binge-eating disorder (BED) has been formally recognized as a distinctive eating disorder. It is characterized by episodes of consuming an unusually large amount of food within a discrete period of time, accompanied by a sense of lack of control over eating, without the recurrent use of inappropriate compensatory behaviors as in bulimia nervosa. As a consequence of such deranged eating, most of the people affected by BED are also obese.

Binge eating can be conceptualized as the result of an excessive motivation to eat. This may arise from the occurrence of either an extreme negative energy balance, as happens in prolonged starvation [2], or the urgent desire to eat for pleasure and/or to reduce anxiety and negative emotions by increasing feelings of pleasure derived from the ingestion of food rather than to fulfill energy needs. In the former case, energy deprivation generates homeostatic hunger, which drives eating in order to remove energy deficit; in the latter case, especially when highly palatable foods are available in the environment, hedonic hunger may generate and drive the consumption of food exclusively to obtain reward from food in spite of no energy need [3]. Accumulating evidence suggests that food-related reward is exerted through brain motivation–reward pathways, and that most of the modulators of homeostatic eating also participate in the control of the rewarding component of food intake [4]. Among those endogenous biochemical mediators, endocannabinoids, i.e., arachidonoylethanolamide (anandamide, AEA) and 2-arachidonoylglycerol (2-AG), have been shown to modulate both homeostatic and rewarding aspects of food intake by stimulating brain cannabinoid CB1 receptors [5,6].

Published studies have shown that overproduction of peripheral endocannabinoids may increase food intake [7] and modulate hedonic eating in both normal weight healthy volunteers and individuals with obesity [8,9]. Furthermore, endocannabinoid CB1 receptors have been shown to play an important role in a rat model of binge eating [10]; however, the role of these mediators in the modulation of hedonic eating in BED has not been explored so far. Since, as stated above, hedonic eating may promote binge eating, understanding the biochemical mechanisms that may mediate the consumption of food for pleasure may increase the knowledge of the pathophysiology of binge eating and help to contrast this pathological behavior. Therefore, in the present study, we assessed plasma endocannabinoid responses to hedonic eating in individuals with BED.

## 2. Materials and Methods

### 2.1. Participants

Patients consecutively admitted to the Eating Disorder Center of the Department of Psychiatry of the University of Campania “Luigi Vanvitelli” of Naples and the Eating and Weight Disorders inpatient unit of Villa Garda Hospital were enrolled into the study if they met the following inclusion/exclusion criteria: (a) diagnosis of BED according to DSM-5 criteria [1]; (b) age ≥ 18 years; (c) no current use of hormones or drugs; (d) no current dieting; (e) absence of severe physical or psychiatric disorders. Seven male patients (mean ± SD age: 36.7 ± 10.1 years; range: 23–55 years) with body mass index ranging from 30.4 kg/m^2^ to 54.0 kg/m^2^ (mean ± SD = 44.18 ± 8.0 kg/m^2^) were recruited. They were tested before starting any specific weight-loss program and signed a written informed consent.

### 2.2. Ethics Statement

The study was approved by the Ethics Committee of the University of Campania “Luigi Vanvitelli” (past Second University of Naples) (ethical approval code: Prot.100; 8 October 2014) and all the procedures were in accordance with the Helsinki Declaration of 1975 as revised in 1983.

### 2.3. Experimental Procedures

Each participant underwent two experimental test-meal sessions, one week apart. The test-meal protocol is largely described elsewhere [8,9]. Briefly, the day before the first experimental session each participant indicated his/her favorite food, that is the food that he/she would eat for pleasure when also satiated. On the first test session, subjects received a breakfast of 300 kcal (including semi-skimmed milk, bread and marmalade or honey with 77% carbohydrates, 10% proteins, and 13% fat) at 9.00 a.m. and rated their hunger and satiety on visual analogue scales (VAS) immediately before and after breakfast. After 1 hour, subjects were told that they would receive their favorite food, and an intravenous catheter was inserted into an antecubital vein to collect a first blood sample (T = 0); then the catheter was connected to a saline solution, which was slowly infused to keep it patent. Immediately afterwards, participants were exposed to the chosen favorite food for 5 min (during this time they could smell and see the food but could not eat it); they then ate the food ad libitum within 10 min. This time period was chosen to standardize the time of food ingestion and the times of blood-sample collection among the subjects in the two experimental sessions. Further blood samples were drawn immediately after the exposure to the favorite food (T5), and 15 (T30) and 120 min (T135) after eating it. Favorite foods were typical Italian cakes with chocolate or “nutella”. At the end of the session, the amount of favorite food eaten by each participant was measured by weighing the residual food and subtracting it from the initial amount of food provided. On the second test session, each participant ate an amount of a non-favorite food (bread + butter) with the same nutrient composition and an equal quantity of calories of his/her favorite food. To this end, the calorie and nutrient contents of favorite and non-favorite foods were calculated by using the WINFOOD program (Medimatica, Teramo, Italy) except for subjects who ate packaged foods with labels. Participants rated hunger, satiety, urge to eat, pleasantness while experiencing a mouthful of food, and amount of food they would eat, on VAS scales immediately after the exposure to food and before eating it.

Blood was collected in tubes with ethylenediaminetetraacetic acid (EDTA) as the anticoagulant. Plasma levels of AEA and 2-AG were determined by isotopic dilution-liquid chromatography-mass spectrometry as described previously [11]. Endocannabinoid assays of the two test sessions for each subject were run at the same time; intra- and inter-assay coefficients of variation were 2.5 ± 0.3% and 10.1 ± 2.5%, respectively, for AEA; and 3.6 ± 0.5% and 12.1 ± 3.4%, respectively, for 2-AG. The areas under the curve (AUC) of the two endocannabinoids were calculated according to the trapezoidal rule.

### 2.4. Statistics

The BMDP statistical software package [12] was used for data analysis. Differences in the biochemical responses to two isoenergetic meals were analyzed by a mixed model analysis of variance (ANOVA) with repeated measures and the post hoc Tukey’s test. ANOVA with repeated measures was employed to analyze differences between the two meal sessions in VAS scores, calorie and nutrient contents of the two types of food. Correlations between VAS scores and AUCs of endocannabinoids were assessed by means of the Pearson’s correlation test. A level of significance of *p* < 0.05 was used for all data analyses.

## 3. Results

### 3.1. VAS Scores

**Figure 1:**
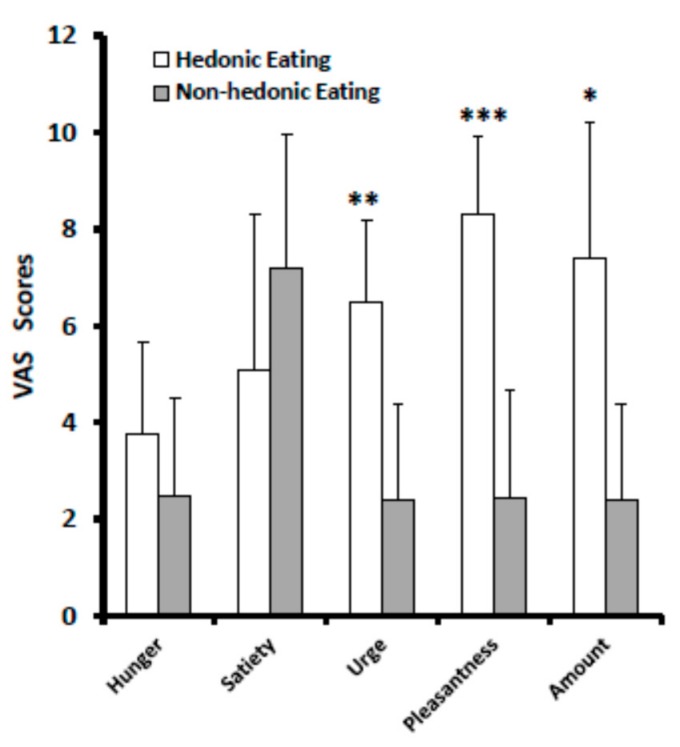
Visual analogue scale (VAS) scores in obese patients with binge-eating disorder in hedonic and non-hedonic eating. Data are expressed as mean ± SD. * *p* = 0.01, ** *p* = 0.007, *** *p* = 0.001 as compared to non-hedonic eating.

ANOVA with repeated measures showed no statistically significant effect between the two meals in the hunger (*F*_1,6_ = 5.39, *p* = 0.06) and satiety (*F*_1,6_ = 4.18, *p* = 0.08) scores while statistically significant differences emerged in the urge to eat (*F*_1,6_ = 15.60, *p* = 0.007), pleasantness (*F*_1,6_ = 34.27, *p* = 0.001) and amount (*F*_1,6_ = 13.04, *p* = 0.01) scores with values in hedonic eating session significantly higher than those in non-hedonic eating session. These results indicate that participants were in a similar satiety condition before eating either the favorite or the non-favorite food, and that the urge to eat, the pleasantness while eating and the amount of food the subject would eat were significantly higher when eating the favorite food than the non-favorite food (Figure 1).

### 3.2. Calories and Nutrients

No statistically significant differences emerged in the mean values of calories, carbohydrates, proteins and lipids of favorite and non-favorite foods (Table 1).

### 3.3. Plasma 2-AG Levels

**Figure 2:**
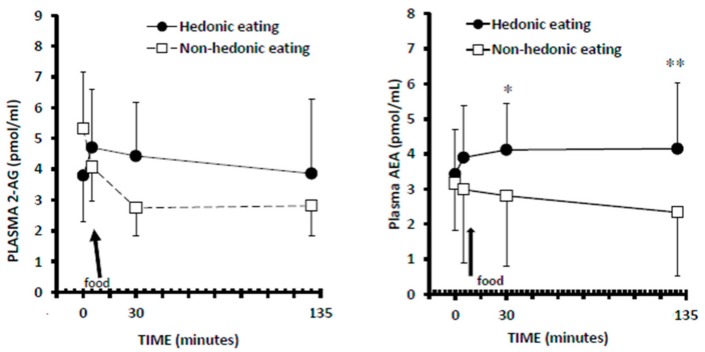
Plasma levels of 2-arachidonoyl glycerol (2-AG) and arachidonoylethanolamide (anandamide, AEA) in hedonic and non-hedonic eating in obese patients with binge-eating disorder. Data are expressed as mean ± SD. The arrows indicate when participants started to eat the test meal. * *p* < 0.05, ** *p* < 0.01 as compared to non-hedonic eating (post-hoc Tukey’s test).

Mixed model 2-way ANOVA with repeated measures showed no significant effect for meal (*F*_1,6_ = 1.28, *p* = 0.3) and for time (*F*_3,18_ = 1.73, *p* = 0.1) and a trend to significant meal–time interaction (*F*_3,18_ = 2.48, *p* = 0.09), indicating that the timing of plasma 2-AG response to favorite food differed slightly and not significantly from that to non-favorite food (Figure 2).

### 3.4. Plasma AEA Levels

Mixed model 2-way ANOVA with repeated measures showed significant effects for meal (*F*_1,6_ = 12.89, *p* = 0.01) but not for time (*F*_3,18_ = 0.21, *p* = 0.8) and trend toward a significant time–meal interaction (*F*_3,18_ = 2.55, *p* = 0.08). Indeed, while plasma levels of AEA progressively decreased after eating the non-favorite meal, they increased after the favorite food (Figure 2). Plasma levels of AEA 15 (T = 30) and 120 min (T = 135) after eating the favorite food were significantly higher than correspondent time point values of non-hedonic eating (Figure 2).

### 3.5. Correlations

Significant positive correlations emerged between the urge-to-eat scores and AUC AEA values (*r* = 0.60, *p* = 0.02), between the pleasantness scores and AUC AEA (*r* = 0.63, *p* = 0.01) or AUC 2-AG values (*r* = 0.63; *p* = 0.01), and between the scores of the amount of food that subjects would eat and AUC 2-AG values (*r* = 0.81, *p* = 0.001).

## 4. Discussions

To the best of our knowledge, this is the first study exploring peripheral endocannabinoid responses to hedonic eating in patients with BED. We found different responses of peripheral 2-AG and AEA to hedonic eating as compared to non-hedonic eating. In particular, plasma levels of AEA progressively decreased after eating the non-favorite food and significantly increased after eating the favorite food; while plasma levels of 2-AG did not differ significantly between the two test conditions, although they showed a trend toward significantly different time patterns, since they progressively decreased in hedonic eating but showed an initial increase followed by a slight decrease in non-hedonic eating. Moreover, the changes in peripheral AEA levels were positively correlated to the subjects’ sensations of the urge to eat and the pleasantness while eating the presented food, while changes in peripheral 2-AG levels were positively correlated to the subjects’ sensation of the pleasantness while eating the presented food and to the amount of food they would eat. These results suggest that, in obese patients with BED, food-induced changes in peripheral endocannabinoids might be associated with the modulation of both the “wanting” and “liking” for food reward, and this may sustain their binge-eating behavior. The mechanisms responsible for such a modulation likely implicate an action of endocannabinoids on central reward pathways, since animal studies have demonstrated that AEA is able to amplify the pleasantness of sucrose taste and to double the number of positive “liking” facial reactions elicited by sucrose taste in rats by acting on nucleus accumbens, a key structure of the central reward system [13,14]. Additionally, blockade of CB1 receptors for endocannabinoids reduces binge-eating behavior also in a rat model [10].

In a previous study with experimental procedures identical to the present ones [9], we found that in individuals with obesity and without BED the plasma levels of 2-AG significantly increased after eating the favorite food, whereas they decreased after eating the non-favorite food; instead, the plasma levels of AEA decreased progressively in non-hedonic eating whereas they showed a reduction after exposure to the favorite food followed by a small but significant increase after eating it. These response patterns seem quite different from those in our BED participants, who exhibited, as compared to our previous obese non-BED subjects, attenuated and not significant changes in 2-AG responses and a clear-cut increase of AEA in hedonic eating. Since both sample groups included individuals with obesity, it seems likely that differences in endocannabinoid responses to hedonic eating are linked to binge eating more than to BMI. In particular, it is tempting to speculate that the less strong changes in 2-AG levels of the present BED participants could be the result of the repeated consumption of large amounts of food during their binge episodes, which may have led to a sort of exhaustion of the 2-AG production with consequent attenuated plasma level response to favorite foods. In agreement with this hypothesis, we observed that, possibly to compensate for the loss of this 2-AG plasma-level response, BED patients exhibited strongly elevated levels of the other endocannabinoid, AEA, following hedonic eating, whereas AEA levels were only slightly increased in our previous patients with obesity and without BED. Furthermore, in our patients with BED, the strongest correlation between plasma endocannabinoid levels and the scores of the urge to eat was in fact found for AEA and not 2-AG levels and this may partly underlie their “wanting” for food. The levels of both compounds correlated with measures of pleasantness, and the levels of 2-AG correlated with the amounts of food eaten which may, therefore, suggest an involvement of both substances in the “liking” rather than the “wanting” for food.

The present results need to be interpreted in light of some limitations. First, in our experimental paradigm participants could eat the food only for 10 min after they were exposed to it; so we cannot exclude the possibility that BED patients would have the pleasure to consume an amount of food greater than that they were allowed to eat, and this could have affected especially the later responses of the endocannabinoids. Indeed, as compared to obese non-binge eaters, obese binge eaters have been found to consume larger amounts of pleasurable food in laboratory conditions [15]. Moreover, the design of the study does not permit us to infer any conclusions about causality between the deranged modulation of food-related reward by endocannabinoids and the development of binge eating. Additionally, we measured plasma levels of AEA and 2-AG; therefore, we cannot conclude that the observed changes in the periphery would occur also in the brain, where the modulation of food-reward by endocannabinoids likely takes place; although, peripheral endocannabinoids have also been suggested as potentially controlling ghrelin release and hence possibly affecting reward [16]. Finally, likely because of the complexity of our experimental paradigm, only seven male patients agreed to participate in the study. The low number of participants with such wide age and BMI ranges and the lack of female subjects could be responsible for false positive results and do not make our results generalizable. Therefore, present findings need confirmation in future studies with larger subject samples that include both male and female BED individuals with obesity.

In summary, in obese patients with BED, hedonic eating was associated with a significant increase of peripheral AEA and slight, although not significant, change in circulating levels of 2-AG as compared to non-hedonic eating. These preliminary findings, together with those obtained in our previously studied obese non-BED individuals, show distinctive responses of endocannabinoids to food-related reward in BED. The relevance of such findings to the pathophysiology of this disorder remains to be elucidated.

## Figures and Tables

**Table 1:** Mean (±SD) calorie and nutrient contents (gram) of favorite and non-favorite foods eaten by participants.

Favorite Food				Non-Favorite Food			
Kcal	Carbohydrates	Proteins	Lipids	Kcal	Carbohydrates	Proteins	Lipids
724.06 ± 381.24	101.60 ± 66.49	12.40 ± 7.80	26.35 ± 16.15	688.97 ± 326.31	91.20 ± 57.52	12.22 ± 9.17	31.77 ± 13.34

No statistically significant difference emerged between the two meals for all the variables (analysis of variance, ANOVA, with repeated measures).
